# Safety culture implications on safe work practices and work place exposure incidents in Operation Theater

**DOI:** 10.12669/pjms.37.2.2946

**Published:** 2021

**Authors:** Tahira Hameed, Eitezaz Ahmed Bashir, Abdul Qadeer Khan, Murtaza Ahmad

**Affiliations:** 1 Dr. Tahira Hameed, MBBS, MRCS. Registrar, Fauji Foundation Hospital, Rawalpindi, Pakistan; 2 Brig. Dr. Eitezaz Ahmed Bashir, FCPS (Surg), Professor and Head General Surgery, Fauji Foundation Hospital, Rawalpindi, Pakistan; 3 Dr. Abdul Qadeer Khan, MBBS, Medical Officer, THQ, Murree, Pakistan; 4 Dr. Murtaza Ahmad, MBBS, Resident Surgery, Sir Ganga Ram Hospital, Lahore, Pakistan

**Keywords:** Culture, Health care, Needle stick injuries, Safety management, Work place

## Abstract

**Background & Objectives:**

The study was undertaken to estimate the prevalence of body and blood fluid exposure incidents such as needle stick injuries, direct contact, splashes, and cuts among health care personnel (HCP) in operation theaters (OTs). The study objective was to investigate perception of safety culture and potential challenges faced by HCP in Pakistani context.

**Methods:**

An analytical cross-sectional survey was conducted in four tertiary care hospitals of Rawalpindi and Islamabad that were Akbar Niazi Teaching Hospital (ANTH), Fauji Foundation Hospital (FFH), Benazir Bhutto Hospital (BBH) and Holy Family Hospital (HFH) during March, 2019 to June, 2019. The data of the current study was collected from surgical staff in OTs at four tertiary care teaching hospitals including nurses, house officers, post graduate trainees, registrars, consultants, and technicians in the twin cities i.e., Rawalpindi and Islamabad.

**Results:**

In our findings (*N=367*) there was a high prevalence of exposure incidents in past six months, 45% had had got a needle injury, 36% have got a splash, 28.8% had direct contact and 35.6% had a cut once respectively in past six months, the occurrence of exposure incidents twice, thrice and more is also enormous.

**Conclusion:**

The study reflected a dire need of trainings at hospitals so that very strategically the importance of safety being a priority and value of HCP is inculcated on daily basis.

## INTRODUCTION

In hospitals there are heightened chances for hospital acquired infections majorly because of clinically compromised state of patients admitted; during their stay, care, communication, and treatment; healthcare personnel (HCP) become prone to various communicable diseases and get exposed to biological hazards.^1^,^2^ HCP can have occupational accidents in the form of percutaneous and mucocutaneous injury, or blood contact with damaged skin through needles, sharps, splashes of blood and other body fluids into eyes, nose, or mouth.^3^,^4^ Over 59 million HCP across the globe are prone of getting infected with AIDS, hepatitis, and tuberculosis by accidentally contaminating with patients’ blood and body fluids. This global issue is overlooked, poorly prevented, and pronounce in developing nations.^5^ Operation theatres (OTs) are the hospital engine room delivering high volume and fast paced procedures.^6^ Safety culture in OTs is intended so that systems, and processes are channeled where potential risk factors are reduced in OTs.

Developing nations like Pakistan face numerous issues such as limited resources and meagre budget to ensure basic health provision and to monitor the biohazards. The surgeons practicing in the system are burdened over un-quantified risk exposure.^7^ The objectives of the study are to estimate the prevalence of exposure incidents in HCP in OTs; to understand perception about safety culture and to explain the challenges faced by HCP in OTs.

## METHODS

After getting ethical approval (Ref. No. FFH/SURG/15/2019 dated 25 January, 2019) from the concerned hospital authority this analytical cross sectional survey was conducted in four tertiary care hospitals that were Akbar Niazi Teaching Hospital (ANTH) located in Islamabad, Fauji Foundation Hospital (FFH), Benazir Bhutto Hospital (BBH), Holy Family Hospital (HFH) located in Rawalpindi during March, 2019 to June, 2019. A total of 500 respondents were approached for survey. The participants of the study were made aware about the nature of the study and their informed consent was obtained; they were assured that data collected will be kept confidential and used only for research purpose. The data was collected by using convenience sampling technique. The participants were provided with a questionnaire booklet that was designed on the basis of existing literature review and group discussions which incorporated sample demographics Form, questions pertaining prevalence of exposure incidents, probable cause, and incident reporting, the last section of the survey comprised of open ended questions on perception of knowledge on safety culture and challenges in carrying out safer work practices in OTs. The surveys took no longer than 15 minutes to be filled in. The quantitative data was entered and analyzed in IBM SPSS version 23, whereas thematic analysis was carried out on qualitative data.

## RESULTS

Among 500 participants, the response rate was 73% as 367 participants (males: *n*=198; females: *n*=169) volunteered to complete the survey Forms with an age range of 21-63 years (*Mean*=31; *SD*= 3.6). 52.8% participants were from private sector *n*= 194 (ANTH: *n*= 40; FFH: *n*= 154) and 47.1 % were from public sector *n*= 173 (BBH: *n*= 125; HFH: *n*= 48).

The participants including nurses, house officers, residents, registrar, consultants and OT technicians were 17%, 40%, 22%, 6%, 3% and 12% respectively. Mostly participants, 117 (31.8%) had less than 1-year work experience and 132 (35.9%) had 1-5 years’ work experience. When studied working hours per week, mostly 105 (28.6%) participants had 60-79 hrs./week working hours and 88 (24%) had above 100 hrs./week working hours.

**Fig.1:**
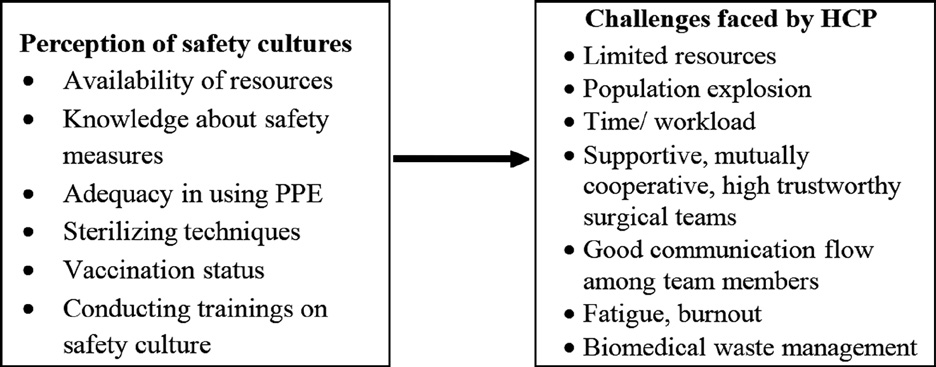
Conceptual framework regarding perception of safety culture and challenges faced by HCP in OTs.

During last six months, needle stick injuries and splashes were higher 45.5% and 47% respectively among HCP. Blood and body fluid exposure multiple times were attributed to factors like stress and long working hours. The incidence of needle stick injuries and cuts twice was 15.5%, 16.6% and 14.4%, and 35.6%, 21.9%, 15.1% respectively. The incidence of needle stick injuries and cuts thrice or more declines depicting the positive attitude in efficacy of precautionary measures taken by HCP. Splashes and direct contact basically have a variation in their incidences because splashes occur accidently and direct contacts are also dependent upon the prevalence of the cases in that particular area. There is significant negative relationship between work experience and exposure to blood and body fluids.

Significant positive correlation between working hours and exposure incidents. The more the working hours the more are the chances of exposure incidents as stress makes the HCP not to work effectively. Table-II. This incidence merely is not that simple because increase in work hours indicating negligence and lack of precautionary measures taken which resulted in higher exposure. The more specific factor attributed to this causation is fatigue, cognitive impairment, restlessness and exertion.

**Table-I:** Relationship between blood, body fluid exposure incidents and work experience (*N*=367).

*Variables*		*1*	*2*	*3*	*4*	*5*
1	Working Experience	-	-0.14*	-0.59**	-0.21*	-0.33*
2	Needle Stick Injuries		-	0.35**	0.38**	0.27**
3	Splashes			-	0.52**	0.25**
4	Direct Contact				-	0.32**
5	Cuts					-

* p ≤ 0.05,

** p ≤ 0.01

**Table-II:** Relationship between blood, body fluid exposure incidents and working hours per week (N=367).

*Variables*		*1*	*2*	*3*	*4*	*5*
1	Working Experience	-	0.61**	0.38**	0.43**	0.36**
2	Needle Stick Injuries		-	0.35**	0.38**	0.27**
3	Splashes			-	0.52**	0.25**
4	Direct Contact				-	0.32**
5	Cuts					-

* p ≤ 0.01

## DISCUSSION

Occupational exposure to blood borne diseases is continuing to be a risk factor for hospital staff and surgical staff is even more vulnerable 8-10 On the surgical floor the HCP can contact hospital acquired infections through body and blood fluid exposure incidents, contaminated surfaces, unsterilized instruments, water, air, dust and fomites.^11^,^12^ HCP working in acute care are more vulnerable to injuries and illnesses. In Pakistani context HCP face challenges as they have inadequate resources, heightened burden of patients, long working hours causing chronic fatigue and burnout. HCP cannot deliver safer care until they feel safe and valued at work place.^13^

Workplace injuries and illnesses cause stress, reduce job satisfaction, and increase turnover intentions.^14^,^15^ Safety culture can be created by fostering mutual trust among HCP.^16^ Education about the importance of protective equipment and double- glove procedure should be emphasized in healthcare setup so that protocols are established for altering attitude and behaviors in risky environment.^9^,^17^ Globally legislations and guidelines have paved way in reducing exposure to blood borne pathogens;^18^ but as there are new emerging infectious agents^19^ and minor errors and negligence can result in large consequences. Systematic and targeted efforts in promoting safety and use of personal protective equipment make difference. Dissemination of knowledge through seminars, workshops, and even practical demonstrations about personal protective equipment, the effectiveness of scrubbing, disinfection, cleaning, aerosolizing of OTs and the OT staff is highly relevant and valid. New methodologies for training of HCP like simulation training and teamwork training appears to be critical for house officers, trainees, registrars, surgeons and all OT staff. We aim to provide training on non-technical levels as well such as communication, teamwork, leadership and decision making that is mandatory in providing optimal health care set up with minimal exposure to bio hazards.

**Graph.1:**
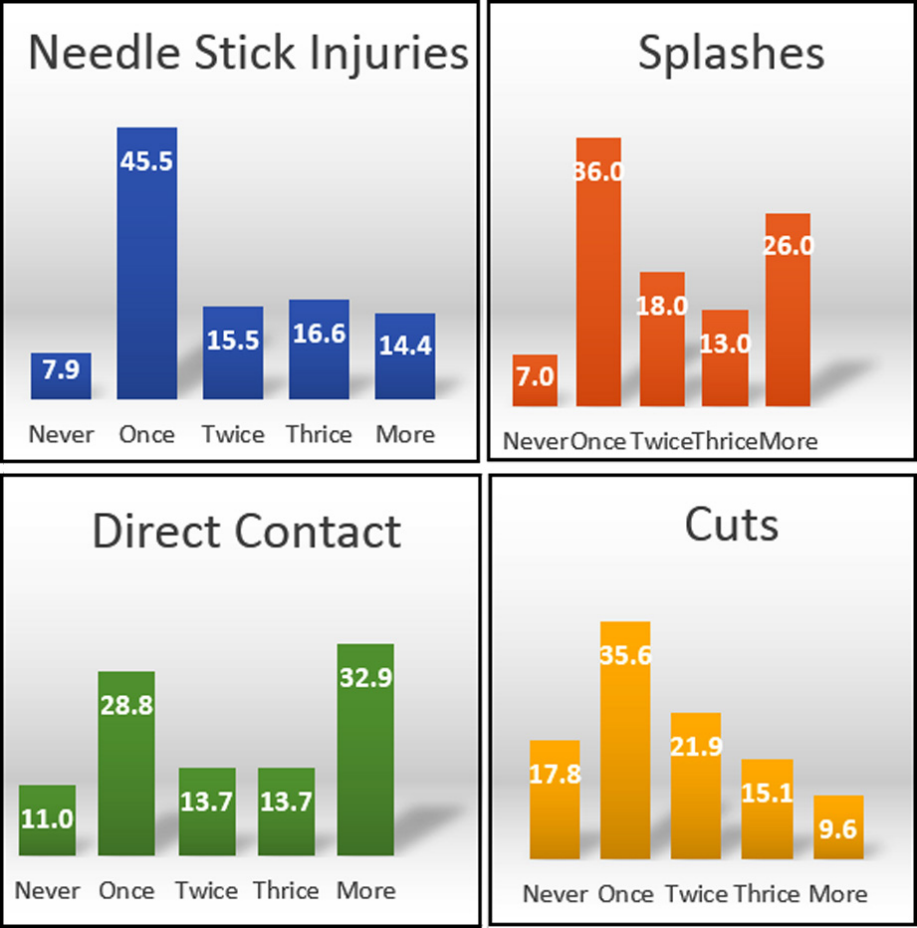
Prevalence of blood, body fluid exposure incidents percentages in HCP in past six months (*N*=367).

### Limitations of the study

It was a cross sectional study that limits answering cause and effect relationships; the data of the study was collected from twin cities of Pakistan therefore limiting the generalizability to rural and small healthcare settings. This study has highlighted the existing problems and challenges faced by the healthcare workers so the various stakeholders need to take measures to overcome the problems.

## CONCLUSION

The current study has highlighted the significance of safety culture as the core value of healthcare sector, the hospital employees when are unaware of efficacy of use of personal protective equipment, have less knowledge of safety culture and dire importance of taking precautionary measures, scrubbing, disinfection, and hygiene of operation theaters, they are more prone to illness, infection, and disease within the staff and cross to the patients as well. So, in Pakistani setup there is need for regulation of work hours and inculcation of safety as integral part of curriculum and practice. When healthcare personnel feel safe and secure, they can ensure sound health delivery with maximum output.

### Author`s Contribution:

**TH** provided concept/research design and did data collection, subjects & editing of manuscript.

**AQK and MA** did statistical analysis and manuscript writing.

**TH and EAB** did editing of manuscript and project management.

**TH** did data collection, subjects and provision of facilities/equipment.

**TH** takes the responsibility and is accountable for all aspects of the work in ensuring that questions related to the accuracy or integrity of any part of the work are appropriately investigated and resolved.
